# Differences in hepatocyte-related indicators within occupational hazardous factor exposure between genders

**DOI:** 10.3389/fpubh.2026.1810652

**Published:** 2026-06-12

**Authors:** Kai Wen, Xu Liao, Xin Huang, Tiecheng Zhang, Zhihao Lin, Yingli Liu, Yanbing Leng

**Affiliations:** 1 Department of Public Health, Chengdu Medical College, Chengdu, Sichuan, China; 2 Nan’an District Center for Disease Control and Prevention, Chongqing, China; 3 Nanchong Mental Health Center of Sichuan Province, Nanchong Second People’s Hospital, Nanchong Senior Hospital, Nanchong, Sichuan, China; 4 Intelligent Perception and Control Key Laboratory of Sichuan Province, Sichuan University of Science and Engineering, Yibin, China

**Keywords:** gender, liver enzymes, manufacturing workers, occupational hazards, propensity score matching

## Abstract

**Objective:**

This study examines how gender differences in exposure to workplace hazards affect liver function markers among manufacturing workers, particularly focusing on physical and chemical exposures such as organic solvents and heavy metals.

**Methods:**

The research draws on data from 1,554 manufacturing workers in China, sourced from the Occupational Health Information Registration System (OHIR). To account for confounding variables like age, body mass index (BMI), and exposure duration, propensity score matching (PSM) was employed. The final analysis involved a balanced sample of 752 workers (376 men and 376 women) to compare liver enzyme levels. Statistical tests, including t-tests, chi-square tests, and conditional logistic regression models, were applied to evaluate these differences.

**Results:**

The results revealed notable gender disparities in both occupational exposure and liver enzyme levels. Male workers were more likely to encounter physical hazards such as dust, heat, and noise, while female workers had a higher exposure to organic chemicals. In terms of liver function, males exhibited significantly higher levels of ALT, AST, GGT, and ALP, with the most marked differences observed in ALT, AST, and GGT levels.

**Conclusion:**

These findings underscore the necessity of factoring in gender when shaping occupational health policies. The study calls for gender-specific interventions to mitigate the risks to liver health, particularly in the manufacturing sector.

## 1 Introduction

Occupational health risks, especially those related to hazardous exposures in manufacturing, have long been a critical concern for workforce safety (1). While much attention has been paid to the health impacts of physical hazards such as noise and dust, the gendered nature of these risks remains underexplored. Are women and men in similar roles truly exposed to the same occupational hazards? (2). And if not, what are the implications for their health, especially concerning liver function, which is often an overlooked indicator of occupational disease (3). We seeks to uncover the complex gender differences in occupational exposure and hepatocyte-related indicators, shedding light on an important yet under-discussed aspect of workplace health.

Manufacturing, as a cornerstone of the global economy, inherently carries a multitude of health risks for its workers. From physical dangers like noise, heat, and dust to chemical exposures involving toxic substances, the potential for occupational diseases is high (4). One of the most concerning outcomes of such exposure is hepatotoxicity, particularly from prolonged contact with chemicals like organic solvents and heavy metals. This type of damage is particularly alarming due to its slow onset and the fact that it often goes unnoticed in its early stages (5). It’s well known that liver function is closely tied to these types of occupational exposures, with enzymes such as alanine transaminase (ALT), aspartate transaminase (AST), *γ*-glutamyl transpeptidase (GGT), and alkaline phosphatase (ALP) serving as common markers to detect liver cell injury (6).

Gender disparities in occupational health risks have garnered increasing attention, revealing that men and women may not only encounter different hazards but also experience distinct health outcomes as a result. Studies has shown that male workers, especially in heavy industries, are more prone to physical hazards, while female workers tend to be more exposed to chemical risks, particularly organic solvents (6, 7). While gender differences in the recognition of occupational health issues might contribute to these variations, the real root of the problem may lie in the unequal distribution of men and women across different job sectors (8–10). This occupational gender division likely plays a significant role in the differing levels of exposure to various workplace hazards (11–13). Yet, despite this understanding, few studies have fully examined how these gendered exposure patterns impact liver health, especially regarding how such exposure influences liver enzyme markers in men and women differently. This gap in knowledge calls for more in-depth research into how occupational hazards specifically affect liver function in male and female workers.

Although previous studies have examined the link between occupational exposure and liver disease, significant gaps remain in our understanding of how gender plays a role in shaping the nature and severity of these risks (12, 14). Specifically, the distinct ways in which men and women are exposed to chemical and physical hazards—and how these exposures affect liver enzyme levels—are not well understood. While it is established that male workers tend to face greater risks from physical hazards like dust, heat, and noise, and that women are often exposed to more organic chemicals, the precise impact of these differing exposures on liver function has yet to be fully clarified (6, 15, 16). This study seeks to address these gaps by exploring how various occupational hazards influence liver enzyme levels, with a particular focus on gender differences within a large cohort of manufacturing workers.

This study sets out to investigate the gender differences in exposure to occupational hazards and their subsequent effects on liver function markers among workers in the manufacturing sector. In particular, the research addresses several key questions: (1) How can propensity score matching (PSM) be applied to adjust for potential confounding variables when examining gender-based exposure patterns? (2) What are the differences in physical and chemical exposure between male and female workers? (3) How do liver enzyme levels vary by gender, and what role do occupational exposures play in these differences? Through the use of PSM, the study aims to offer a clearer comparison of gender-based liver function disparities, controlling for factors such as age, body mass index (BMI), and the duration of exposure to occupational hazards.

## 2 Methods

### 2.1 Study population

**Figure 1:**
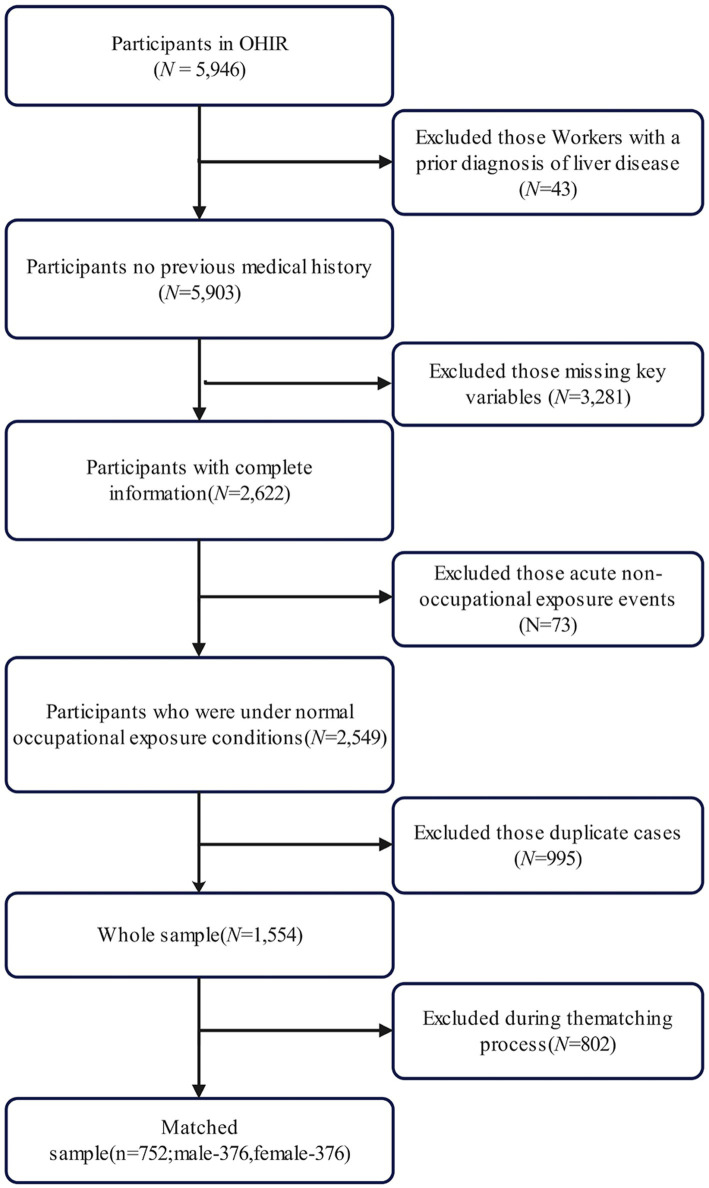
Flow diagram of study population selection from OHIR.

This study is based on data from the Occupational Health Information Registration System (OHIR) of Nanan District, Chongqing, which includes 5,946 active manufacturing workers who underwent occupational health check-ups between 2023 and 2024. The inclusion criteria were: (1) on-duty manufacturing workers aged ≥18 years; (2) documented exposure to specific occupational hazard factors; (3) at least one liver function test, with priority given to expert review if there is a significant discrepancy between the latest and previous test results; and (4) no history of liver disease. Among these participants, we excluded those with a prior diagnosis of liver disease (*n* = 43). We also excluded participants with missing key variables (*n* = 3,281), acute non-occupational exposure events (*n* = 73), and duplicate cases (*n* = 995). The final analysis included 1,554 manufacturing workers. To investigate gender differences within the same occupations, we applied a one-to-one matching process, resulting in a subset of 752 participants from the overall sample (Figure 1). This study was approved by the Ethics Committee of the Chongqing Nanan District Center for Disease Control and Prevention.

### 2.2 Hepatocyte injury indicators

The outcome variable for hepatocyte injury was recorded as a binary variable (Yes/No). Hepatocyte injury was determined based on four serum enzyme indicators: alanine transaminase (ALT), aspartate transaminase (AST), γ-glutamyl transpeptidase (GGT), and alkaline phosphatase (ALP). According to the 10th edition of “Diagnostics,” the clinical reference ranges for these enzymes are as follows: ALT (male: 5–40 U/L, female: 5–35 U/L); AST (male: 8–40 U/L, female: 8–35 U/L); GGT (male: 11–50 U/L, female: 9–40 U/L); ALP (45–125 U/L) (17). Hepatocyte injury is defined when either ALT or AST exceeds the corresponding upper limit of normal (ULN). GGT and ALP are included in this study as auxiliary indicators to aid in the interpretation of the type of liver injury.

Occupational health examinations were performed by medical personnel who had received standardized training. The collected general information from workers included their name, age, gender, height, weight, exposure factors, duration of exposure, medical history, and smoking.

### 2.3 Classification of occupational exposure

Occupational exposure information was obtained through a standardized occupational health questionnaire and factory occupational health records. Chemical exposure was defined according to known hepatotoxic substances (e.g., organic solvents, heavy metals) listed in GBZ 59—2024 “Diagnostic Criteria for Occupational Toxic Liver Diseases” (18).

### 2.4 Statistical analysis

Data analysis was conducted using R software (version 4.5.2). To assess the impact of occupational category differences on gender disparities, the study employed propensity score matching (PSM), considering factors such as age, education, and employment status. Each male worker was matched one-to-one with a female worker from the same occupation. Standardized mean differences (SMD) were calculated to evaluate the balance between the matched groups, with an SMD value below 0.25 indicating acceptable differences, ensuring the reliability of gender comparisons.

Following the matching process, *t*-tests were conducted to analyze liver cell injury indicators, while chi-square or Fisher’s exact tests were used to compare gender differences in liver injury markers. To further explore gender differences in occupational hazard exposure, a conditional logistic regression model was employed, adjusting for covariates like age and education. Results were reported as odds ratios (*OR*) with 95% confidence intervals (*CI*). All statistical tests were two-sided, and statistical significance was set at *p* < 0.05. Additionally, an alternative matching analysis using Mahalanobis distance was performed to further validate the robustness of the propensity score matching method.

## 3 Results

### 3.1 Basic demographic of gender differences in occupational and health

This research employed propensity score matching (PSM) to examine gender disparities within both the full sample (378 females, 1,176 males) and the matched sample (376 females, 376 males). At the outset, the average age for females was 35.07 ± 9.93 years, while males averaged 37.16 ± 11.26 years, yielding a standardized mean difference (*SMD*) of 0.191. After matching, the *SMD* dropped to 0.0898, effectively mitigating age-related discrepancies. Regarding body mass index (BMI), females had an average BMI of 22.64 ± 3.17, while males had 23.51 ± 3.36, producing an *SMD* of 0.263.

After matching, the *SMD* decreased to 0.0942, suggesting that the BMI disparities were well-adjusted. Regarding years of occupational exposure, females had an average of 1.82 ± 3.02 years, while males averaged 1.85 ± 3.27 years, resulting in an *SMD* of 0.00881. Post-matching, the *SMD* was 0.0179, indicating that gender differences remained minimal. In terms of workplace size distribution, 3.70% of females were employed in micro-sized units, and 42.06% in small-sized units, while the respective figures for males were 5.19 and 52.13%. Following matching, the *SMD* reduced to 0.0186, significantly narrowing the gender gap in workplace size distribution. As for employment status, 53.17% of females had left their jobs prior to the study, with 36.24% still employed, compared to 63.44 and 30.02% for males, respectively. After matching, the *SMD* for employment status decreased to 0.0372, reflecting effective adjustment of gender differences. Lastly, smoking status showed a significant gender disparity in the full sample (*SMD* = 0.616), but following matching, the *SMD* dropped to 0.000, indicating a complete elimination of gender differences in smoking behavior (see Table 1).

**Table 1:** Distribution of gender differences in occupational health and demographic variables.

Variables	Whole sample			Matched sample		
	Female (*n* = 378)	Male (*n* = 1,176)	Standardized mean difference	Female (*n* = 376)	Male (*n* = 376)	Standardized mean difference
	*N*(%)	*N*(%)		*N*(%)	*N*(%)	
Age, mean ± SD	35.07 ± 9.93	37.16 ± 11.26	0.191	35.15 ± 9.91	35.64 ± 11.40	0.0898
BMI, mean ± SD	22.64 ± 3.17	23.51 ± 3.36	0.263	22.66 ± 3.17	23.09 ± 3.31	0.0942
Years of hazardous work, mean ± SD	1.82 ± 3.02	1.85 ± 3.27	0.00881	1.83 ± 3.02	2.01 ± 3.27	0.0179
Employment unit size			0.261			0.0186
Micro	14 (3.70)	61 (5.19)		147 (39.10)	144 (38.30)	
Small	159 (42.06)	613 (52.13)		159 (42.29)	163 (43.35)	
Medium	56 (14.81)	179 (15.22)		14 (3.72)	16 (4.26)	
Large	149 (39.42)	323 (27.47)		56 (14.89)	53 (14.10)	
Employment Status			0.0380			0.0372
After	40 (10.58)	77 (6.55)		38 (10.11)	35 (9.31)	
Before	201 (53.17)	746 (63.44)		201 (53.46)	217 (57.71)	
During	137 (36.24)	353 (30.02)		137 (36.44)	124 (32.98)	
Smoking Status			0.616			0.000
No	376 (99.47)	902 (76.70)		374 (99.47)	374 (99.47)	
Yes	2 (0.53)	274 (23.30)		2 (0.53)	2 (0.53)	

### 3.2 Comparison of liver enzyme levels between genders

**Figure 2:**
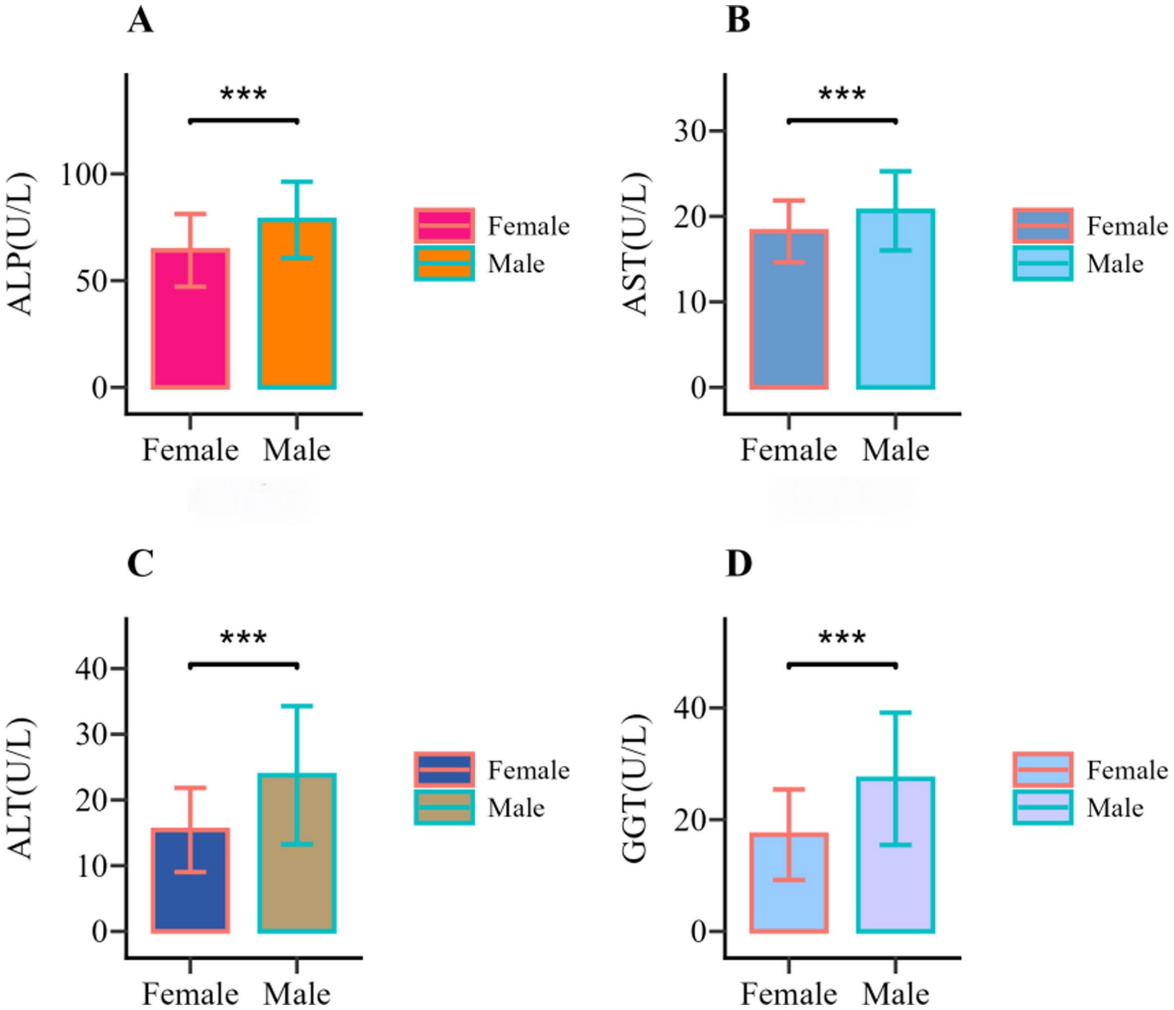
Gender differences in hepatocyte-related indicators. **(A)** ALP; **(B)** AST; **(C)** ALT; **(D)** GGT. Data are presented as median values with interquartile ranges. Significant differences between genders are indicated by *p < 0.05, **p < 0.01, and ***p < 0.001.

The examination of liver enzyme levels—ALT, AST, GGT, and ALP—revealed striking gender differences, the details (see Figure 2). For ALT, the median value in females was 13.8 (IQR: 11.4–17.8), while males showed a notably higher median of 21.6 (IQR: 15.8–29.5), with the difference proving statistically significant (*p* < 0.001). A similar trend emerged for AST: females had a median of 17.7 (IQR: 15.7–20.3), and males had a higher median of 20.0 (IQR: 17.4–23.4), again with a significant *p*-value (*p* < 0.001). Regarding GGT, females had a median of 15.0 (IQR: 12.1–19.0), while males had a markedly elevated median of 25.1 (IQR: 19.0–34.3), with a *p*-value of <0.001, further underscoring the gender disparity in this enzyme. Lastly, for ALP, the median for females was 61.0 (IQR: 52.0–73.2), compared to 77.0 (IQR: 66.0–90.0) for males, a difference that was also statistically significant (*p* < 0.001).

### 3.3 Differences in liver enzyme marker levels

For ALT, a small percentage of both females (3.73%) and males (4.17%) had elevated levels, with no significant gender difference observed (*χ^2^* = 2.54, *p* = 0.111). In contrast, AST levels were higher in females (6.50%) than in males (7.65%), with a statistically significant difference (*χ^2^* = 10.59, *p* = 0.001), suggesting that males had a higher prevalence of elevated AST. GGT levels, however, showed similar proportions of elevated values between females (2.06%) and males (2.21%), and no significant difference was detected (*χ^2^* = 0.55, *p* = 0.458). Lastly, for ALP, a higher percentage of females (2.38%) had elevated levels compared to males (1.79%), with this difference being statistically significant (*χ^2^* = 7.37, *p* = 0.007), indicating a higher prevalence of elevated ALP in females. While gender differences in liver enzyme markers were evident, only AST and ALP showed statistically significant variations (see Table 2).

**Table 2:** Gender differences in liver enzyme levels and proportions of abnormal values.

Variables	Total (*n* = 1,554)	Gender		Statistic	*P*
		Female (*n* = 378)	Male (*n* = 1,176)		
ALT mark, *n*(%)				*χ^2^* = 2.54	0.111
No	1,496 (96.27)	369 (97.62)	1,127 (95.83)		
Yes	58 (3.73)	9 (2.38)	49 (4.17)		
AST mark, *n*(%)				*χ^2^* = 10.59	0.001
No	1,453 (93.50)	367 (97.09)	1,086 (92.35)		
Yes	101 (6.50)	11 (2.91)	90 (7.65)		
GGT mark, *n*(%)				χ^2^ = 0.55	0.458
No	1,522 (97.94)	372 (98.41)	1,150 (97.79)		
Yes	32 (2.06)	6 (1.59)	26 (2.21)		
ALP mark, *n*(%)				*χ^2^* = 7.37	0.007
No	1,517 (97.62)	362 (95.77)	1,155 (98.21)		
Yes	37 (2.38)	16 (4.23)	21 (1.79)		

*χ*^2^, Chi-square test.

### 3.4 Differences in occupational hazard exposure

In the full sample, male workers had significantly higher exposure rates to physical hazards such as dust, heat, and noise. Males were more likely to be exposed to dust (*OR* = 2.40), heat (*OR* = 2.03), and noise (*OR* = 2.40), with all differences showing statistical significance (*p* < 0.001) (see Table 3). Regarding chemical exposures, males had a higher likelihood of encountering inorganic chemicals, although the difference was not significant, while females were more likely to be exposed to organic chemicals (*OR* = 0.53, *p* < 0.001). In the matched sample, similar trends were observed. Males continued to report higher exposure to physical hazards, including dust (*OR* = 2.82), heat (*OR* = 2.05), and noise (*OR* = 3.18), with all differences remaining statistically significant. For chemical exposures, males showed greater exposure to inorganic chemicals (*OR* = 2.61, *p* < 0.001), but no significant difference was observed for organic chemicals (*OR* = 1.23, *p* = 0.441). The sensitivity analysis, performed with an alternative matching method, corroborated the primary results (see Supplementary Table S1). After the matching process, male workers continued to exhibit a higher prevalence of physical, chemical, and biological hazards compared to female workers. Potential confounding factors are presented in Supplementary Table S2.

**Table 3:** Gender differences in occupational hazard exposure across physical and chemical risks.

Occupational hazards	Whole sample			
	Male (%)	Female (%)	*P*-value	OR (95% CI)
Physical factors				
No	127 (33.60)	207 (17.60)		1.00 (Reference)
Dust exposure	22 (5.82)	86 (7.31)	<0.001	2.40 (1.43 ~ 4.03)
Heat exposure	19 (5.03)	63 (5.36)	0.013	2.03 (1.16 ~ 3.56)
Noise exposure	210 (55.56)	820 (69.73)	<0.001	2.40 (1.83 ~ 3.13)
Chemical factors				
No	225 (59.52)	804 (68.37)		1.00 (Reference)
Inorganic chemical exposure	44 (11.64)	164 (13.95)	0.82	1.04 (0.72 ~ 1.50)
Organic chemical exposure	109 (28.84)	208 (17.69)	<0.001	0.53 (0.41 ~ 0.70)
Matched sample				
Physical factors				
No	125 (33.24)	71 (18.88)		1.00 (Reference)
Dust exposure	22 (5.85)	23 (6.12)	0.009	2.82 (1.30 ~ 6.13)
Heat exposure	19 (5.05)	22 (5.85)	0.049	2.05 (1.01 ~ 4.19)
Noise exposure	210 (55.85)	260 (69.15)	<0.001	3.18 (1.87 ~ 5.42)
Chemical factors				
No	225 (59.84)	249 (66.22)		1.00 (Reference)
Inorganic chemical exposure	44 (11.70)	62 (16.49)	<0.001	2.61 (1.48 ~ 4.62)
Organic chemical exposure	107 (28.46)	65 (17.29)	0.441	1.23 (0.73 ~ 2.07)

OR, odds ratio; CI, confidence interval.

## 4 Discussion

This study aimed to explore gender differences in occupational hazard exposures and hepatocyte-related indicators among manufacturing workers. The results highlight significant gender disparities in both physical and chemical exposures, as well as in liver enzyme levels. Males were more likely to be exposed to physical hazards, such as dust, heat, and noise, while females showed greater exposure to organic chemicals. These findings have important implications for understanding gender-specific occupational health risks.

The results indicate that male workers were significantly more likely to experience physical hazards, with exposure rates to dust, heat, and noise being considerably higher than their female counterparts. These findings suggest that male workers, especially in industries like manufacturing, may face higher risks of physical harm (19). It is established that prolonged exposure to these physical hazards, particularly noise, is associated with long-term health problems, such as hearing loss and respiratory issues (2, 20, 21). Thus, targeted interventions aimed at reducing physical hazards for male workers are crucial. In contrast, females in this study were more likely to report exposure to organic chemicals (22, 23). Across the unmatched sample, female workers had more frequent organic-chemical exposure, but this difference weakened after matching. This pattern may reflect gender-based job allocation rather than an inherent sex difference. Women may be assigned to tasks such as cleaning, coating, printing, packaging, inspection, or laboratory quality control, where exposure to solvents such as benzene, toluene, xylene, n-hexane, acetone, ethyl acetate, trichloroethylene, and dichloromethane may occur. Several of these agents are hepatotoxic (24). A more detailed characterization of solvent exposure by specific task could offer a stronger explanation for the gender-related differences observed in liver enzyme profiles. The observed difference, with females being 0.53 times less likely to be exposed to these chemicals, suggests a distinct gendered exposure pattern in the chemical risks faced by workers. It has been suggested that certain job roles predominantly occupied by women, such as administrative positions in laboratories or quality control, may expose them to organic chemicals more frequently than their male counterparts (25).

The higher median levels of ALT, AST, GGT, and ALP observed in male workers are better regarded as early indicators of hepatic burden, rather than as direct evidence of occupational toxic liver disease (24). This interpretation is supported by the low overall proportion of abnormal enzyme values, despite statistically significant differences between genders. Such a pattern is more consistent with possible subclinical hepatocellular stress than with overt liver injury (26). Mild increases in aminotransferases and GGT have also been reported in workers exposed to organic solvents, metals, or mixed occupational hazards, suggesting that liver enzymes may reflect an early biological response to occupational exposure (6, 27). These markers, however, are not specific to occupational liver injury; they may also be influenced by metabolic status, lifestyle factors, and individual susceptibility (28). The present findings therefore provide a preliminary basis for understanding gender-related hepatic responses, while longitudinal evidence is still needed to determine their long-term health significance.

These sex-specific hepatic patterns also be viewed through the liver–spleen immunometabolic axis. The liver remains the primary organ in which industrial toxicants are metabolized and detoxified, yet the subsequent inflammatory tone is not determined by the liver alone (29). Through immune-cell trafficking, cytokine release, and removal of blood-borne inflammatory stimuli, the spleen may condition the hepatic response to occupational stressors (30). On this basis, dimorphic splenic immune regulation could influence how a given exposure is expressed as hepatocellular strain. Estrogen-linked anti-inflammatory activity may blunt secondary injury in women, whereas a more pro-inflammatory milieu under chemical or physical stress may leave male hepatocytes more vulnerable to enzyme leakage (31, 32). The findings of this study align with much of the existing literature that highlights higher exposure to physical hazards among male workers, particularly in manufacturing industries (3, 33, 34). Previous studies have similarly reported that males are disproportionately exposed to physical hazards like noise and dust, which are prevalent in sectors like construction and manufacturing (15, 35, 36). However, this study offers a new perspective by examining gender differences in the exposure to organic chemicals, a relatively underexplored area. The results of this study are consistent with research showing that females tend to have greater exposure to organic solvents in certain roles, which are often linked to liver damage (37–39). From a long-term perspective, chemical hazards may be more directly relevant to liver injury, as organic solvents and metals undergo hepatic metabolism and may trigger oxidative stress, inflammation, and low-grade hepatocellular injury. Physical hazards such as noise, dust, and heat may influence liver-related health more indirectly through systemic stress, respiratory burden, cardiovascular strain, and metabolic dysregulation. Their relative long-term effects should be clarified in longitudinal studies with quantitative exposure assessment.

Additionally, the study’s use of PSM improves upon previous research by reducing potential confounders and allowing for a more accurate comparison between genders (40). This methodological approach strengthens the reliability of the findings and provides a clearer picture of the gendered nature of occupational hazards. From a policy perspective, these findings could inform future occupational health regulations and safety guidelines that specifically address gender differences (41). But, this study has several limitations. Its cross-sectional design prevents establishing causal relationships between occupational exposures and liver function. Longitudinal studies are needed to assess the long-term effects on workers’ health. Additionally, confounding variables, such as job role variations within genders, were not fully addressed. Future research should include detailed job-specific data and consider the role of individual protective measures, which may differ by gender.

## 5 Conclusion

In summary, this study highlights significant gender differences in both physical and chemical exposures in the workplace, with males being more likely to encounter physical hazards and females more frequently exposed to organic chemicals. These findings underscore the need for gender-sensitive occupational health policies and interventions. Future research should focus on investigating the long-term health consequences of these exposures, particularly liver function, and explore more comprehensive methodologies to account for individual job tasks and protective measures in occupational settings.

## Data Availability

The raw data supporting the conclusions of this article will be made available by the authors, without undue reservation.
