# Supplementary material for: Differences in hepatocyte-related indicators within occupational hazardous factor exposure between genders
Source: Front Public Health. 2026 Jun 12;14:1810652. doi: 10.3389/fpubh.2026.1810652 (PMC13303727; doi:10.3389/fpubh.2026.1810652)
Supplement: Supplementary file 1 [file data_sheet_1.docx]

**Supplementary Table 1.** Exposure prevalence of males and females in matched sample with robust-Mahalanobis distance.

| **Variables** | **Total**  **(*n* = 1554)** | **Female**  **(*n* = 378)** | **Male**  **(*n* = 1176)** | ***P*** | ***OR* (95%CI)** |
| --- | --- | --- | --- | --- | --- |
| Physical Factors, *n*(%) |  |  |  |  |  |
| No | 334 (21.49) | 127 (33.60) | 207 (17.60) |  | 1.00 (Reference) |
| Dust Exposure | 108 (6.95) | 22 (5.82) | 86 (7.31) | 0.023 | 2.45 (1.13 ~ 5.28) |
| Heat Exposure | 82 (5.28) | 19 (5.03) | 63 (5.36) | 0.006 | 2.67 (1.33 ~ 5.35) |
| Noise Exposure | 1030 (66.28) | 210 (55.56) | 820 (69.73) | <0.001 | 2.79 (1.70 ~ 4.57) |
| Chemical Factors, *n*(%) |  |  |  |  |  |
| No | 1029 (66.22) | 225 (59.52) | 804 (68.37) |  | 1.00 (Reference) |
| Inorganic Chemical Exposure | 208 (13.38) | 44 (11.64) | 164 (13.95) | 0.026 | 1.82 (1.07 ~ 3.08) |
| Organic Chemical Exposure | 317 (20.40) | 109 (28.84) | 208 (17.69) | 0.777 | 1.07 (0.65 ~ 1.76) |

**Supplementary Table S2.** Regression results for potential confounding factors associated with occupational hazard exposure

| **Variables** | **β** | **S.E** | **Z** | ***P*** | **OR (95%CI)** |
| --- | --- | --- | --- | --- | --- |
|  |  |  |  |  |  |
|  | **Whole sample** | | | | |
| Age | 0 | 0.01 | -0.05 | 0.962 | 1.00 (0.99 ~ 1.01) |
| BMI | 0.09 | 0.02 | 4.16 | <0.001 | 1.09 (1.05 ~ 1.14) |
| Employment Unit Size |  |  |  |  |  |
| Large |  |  |  |  | 1.00 (Reference) |
| Medium | 0.39 | 0.22 | 1.75 | 0.08 | 1.47 (0.96 ~ 2.27) |
| Micro | 0.46 | 0.34 | 1.38 | 0.169 | 1.59 (0.82 ~ 3.08) |
| Small | 0.37 | 0.15 | 2.44 | 0.015 | 1.45 (1.08 ~ 1.96) |
| Employment Status |  |  |  |  |  |
| After Employment |  |  |  |  | 1.00 (Reference) |
| Before Employment | 0.44 | 0.23 | 1.88 | 0.06 | 1.55 (0.98 ~ 2.44) |
| During Employment | 0.01 | 0.25 | 0.06 | 0.954 | 1.01 (0.62 ~ 1.66) |
| Current Smoking Status |  |  |  |  |  |
| Non-smoker |  |  |  |  | 1.00 (Reference) |
| Occasional | 4.21 | 0.72 | 5.88 | <0.001 | 67.31 (16.55 ~ 273.73) |
|  | **Matched sample** | | | | |
| Age | 0 | 0.01 | 0.13 | 0.896 | 1.00 (0.98 ~ 1.02) |
| BMI | 0.04 | 0.02 | 1.5 | 0.133 | 1.04 (0.99 ~ 1.09) |
| Employment Unit Size |  |  |  |  |  |
| Large |  |  |  |  | 1.00 (Reference) |
| Medium | 0.07 | 0.26 | 0.28 | 0.783 | 1.07 (0.65 ~ 1.79) |
| Micro | 0.03 | 0.4 | 0.07 | 0.941 | 1.03 (0.47 ~ 2.27) |
| Small | 0.04 | 0.18 | 0.2 | 0.842 | 1.04 (0.73 ~ 1.47) |
| Employment Status |  |  |  |  |  |
| After Employment |  |  |  |  | 1.00 (Reference) |
| Before Employment | 0.01 | 0.27 | 0.04 | 0.967 | 1.01 (0.60 ~ 1.71) |
| During Employment | -0.01 | 0.28 | -0.04 | 0.97 | 0.99 (0.57 ~ 1.72) |
| Current Smoking Status |  |  |  |  |  |
| Non-smoker |  |  |  |  | 1.00 (Reference) |
| Occasional | -0.08 | 1.05 | -0.08 | 0.937 | 0.92 (0.12 ~ 7.27) |
| OR: Odds Ratio, CI: Confidence Interval | | | | | |
